# Integrin Regulation of CAF Differentiation and Function

**DOI:** 10.3390/cancers11050715

**Published:** 2019-05-24

**Authors:** C. Michael DiPersio, Livingston Van De Water

**Affiliations:** Department of Surgery, Albany Medical College, Albany, NY 12208, USA; vandewl@amc.edu

**Keywords:** integrin, tumor microenvironment, cancer-associated fibroblast (CAF), extracellular matrix, paracrine signaling

## Abstract

Extensive remodeling of the extracellular matrix, together with paracrine communication between tumor cells and stromal cells, contribute to an “activated” tumor microenvironment that supports malignant growth and progression. These stromal cells include inflammatory cells, endothelial cells, and cancer-associated fibroblasts (CAFs). Integrins are expressed on all tumor and stromal cell types where they regulate both cell adhesion and bidirectional signal transduction across the cell membrane. In this capacity, integrins control pro-tumorigenic cell autonomous functions such as growth and survival, as well as paracrine crosstalk between tumor cells and stromal cells. The myofibroblast-like properties of cancer-associated fibroblasts (CAFs), such as robust contractility and extracellular matrix (ECM) deposition, allow them to generate both chemical and mechanical signals that support invasive tumor growth. In this review, we discuss the roles of integrins in regulating the ability of CAFs to generate and respond to extracellular cues in the tumor microenvironment. Since functions of specific integrins in CAFs are only beginning to emerge, we take advantage of a more extensive literature on how integrins regulate wound myofibroblast differentiation and function, as some of these integrin functions are likely to extrapolate to CAFs within the tumor microenvironment. In addition, we discuss the roles that integrins play in controlling paracrine signals that emanate from epithelial/tumor cells to stimulate fibroblasts/CAFs.

## 1. Introduction

**Figure 1:**
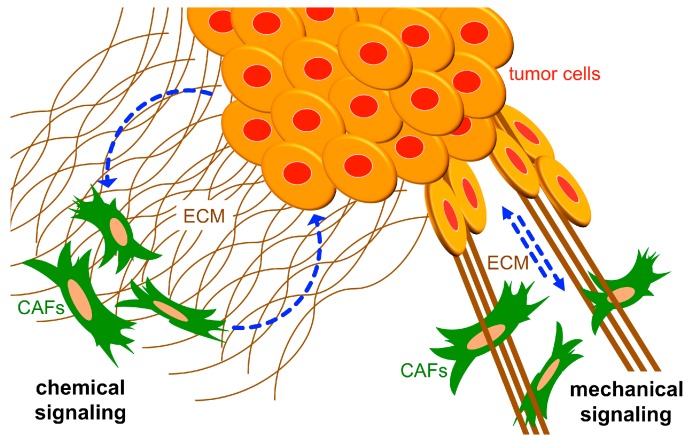
Integrins regulate intercellular communication in the tumor microenvironment (TME). ECM receptors (integrins) on the cell surface (not depicted in the illustration) regulate paracrine signaling between tumor cells and stromal CAFs. Paracrine signaling can be mediated by secreted factors (i.e., chemical signaling, depicted on the left) or changes in matrix stiffness (i.e., mechanical signaling, depicted on the right). ECM: extracellular matrix; CAFs: cancer-associated fibroblasts.

The tumor microenvironment (TME) is an important driver of tumor growth and malignant progression. Within the TME, an “activated” stroma depends on extensive modulation of the extracellular matrix (ECM) and communication between tumor cells and cells that reside in the stroma, including tumor/cancer-associated fibroblasts (CAFs), infiltrating immune and inflammatory cells, and endothelial cells of the tumor vasculature [1,2,3,4]. Integrins are the major family of cell adhesion receptors for the ECM [5], and they are expressed on all cell types of the TME where they have critically important roles in tumor growth and malignant progression. Indeed, integrins regulate a number of cell-autonomous functions within both tumor cells and stromal cells that promote cancer, including survival, proliferation, motility/invasion, and ECM modulation, as reviewed extensively elsewhere [6,7,8,9,10,11]. In addition, roles are emerging for integrins in the regulation of intercellular communication within the TME, through their abilities to regulate paracrine signaling, either chemical or mechanical, between tumor cells and stromal cells [12,13] (Figure 1).

All integrins are heterodimeric, transmembrane glycoproteins consisting of an α and β subunit, each with a cytoplasmic domain, a transmembrane domain, and a large extracellular domain. There are 24 distinct integrins that result from the dimerization of 18 α subunits and eight β subunits in limited combinations, each with different (albeit often overlapping) ligand-binding specificities [5]. As receptors at the cell’s interface with the ECM, integrins bind simultaneously with ligands through their extracellular domains, and with cytoskeletal/signaling proteins through their cytoplasmic domains, thereby mediating both ‘inside-out’ and ‘outside-in’ signaling. In this capacity, integrins are conduits of bidirectional signal transduction that control the cell’s ability to both promote and respond to biochemical and mechanical changes in the TME [5,14]. This regulation reflects the central roles that integrins play in the “dynamic reciprocity” between cells and the ECM that has long been appreciated [15].

As integrins have pro-tumorigenic roles in both tumor and stromal cells, and their cell surface expression renders them readily accessible to inhibitory agents, they are attractive therapeutic targets [7,16]. Indeed, notable clinical successes have been realized with integrin-based therapeutics that target platelet-specific integrin αIIbβ3 (thrombosis), leukocyte integrins α4β1 and α4β7 (multiple sclerosis), α4β7 (ulcerative colitis and Crohn’s disease), and αLβ2 (dry eye) (for recent comprehensive reviews, see [17,18]). However, attempts to exploit integrins as targets in the cancer clinic have so far been met with little success [19,20,21]. The inefficacy of integrin-targeting strategies to date can be attributed in a large part to several important limitations of current approaches. First, these approaches have focused on the use of arginine-glycine-aspartic acid (RGD)-based peptides or RGD mimetics, the first discovered peptide motif within certain ECM proteins (e.g., fibronectin, FN) that binds a small subgroup of integrins [5,7,20]. As a result, integrins that bind non-RGD ligands, such as laminins (LNs) or non-RGD domains of FN, remain unexplored as clinical targets despite abundant evidence from preclinical models that supports important roles in cancer [8,22,23,24,25,26,27,28,29,30]. Second, many integrin antagonists that have been tested in the cancer clinic are thought to work primarily by targeting integrins on endothelial cells of the tumor vasculature to modulate angiogenesis (e.g., αvβ3 or αvβ5) [7]. However, clinical strategies to target integrins on the tumor cells, CAFs, or other stromal cells, remain underdeveloped. Finally, many clinical and preclinical studies of integrin function in cancer have focused on and targeted the β1 integrins as a group, while paying insufficient attention to the broad functional diversity among the twelve distinct αβ heterodimers that comprise the β1 integrin subfamily [5]. These β1 subfamily members can bind different ligands and have distinct roles in cancer. For example, α2β1 is a suppressor of breast cancer metastasis [31], while α3β1 and α6β1 have pro-tumorigenic/pro-metastatic roles in breast cancer [25,32,33,34,35].

The striking discordance between decades of preclinical studies that strongly support important roles for integrins in cancer, and the disappointing results of cancer clinical trials that target integrins, reflect how the field of integrin biology has evolved to a critical juncture at which we must begin to delve more deeply into the combined roles that individual integrin αβ heterodimers play within distinct cell types of the TME. Moreover, in addition to their well-established roles in regulating cell-autonomous functions, there is mounting evidence that some integrins can also regulate communication between different cell types during both normal and pathological tissue remodeling processes, as reviewed elsewhere [12,36,37,38]. These findings link the adhesion state of a cell—which is defined by the function of specific integrins—with its capacity to influence the phenotype of other cells. While this phenomenon is less well explored in cancer, it is likely that integrins have similarly important roles in paracrine signaling and intercellular communication in the TME to support tumor growth and progression. Indeed, as discussed below, there is much we can learn from intercellular communication between epithelial cells and fibroblasts/myofibroblasts during wound healing that may also apply to interactions between tumor cells and CAFs in the TME [39]. 

## 2. Roles for Integrins in Controlling CAF Differentiation and Function

It is well established that CAFs contribute to an activated stroma that promotes tumor progression and carcinoma invasion, and roles for specific integrins in this process are beginning to emerge. In the following discussion, we use the wound myofibroblast as a model for the CAF. Myofibroblasts deposit ECM exuberantly and effect its robust contraction. Indeed, the myofibroblast-like properties of CAFs are important for their ability to generate a stiff ECM within the TME that supports invasive tumor growth [4,40,41]. As discussed below, roles for specific integrin-ECM interactions in controlling myofibroblast differentiation and ECM rigidity are clear [42,43]. Integrins can also regulate the ability of CAFs to modulate the TME by controlling the bioavailability of ECM-bound growth factors, or the exposure/liberation of matrix protein domains/fragments with bioactivity through mechanical signaling or ECM proteolysis [44,45,46]. Well known examples include the local activation of the ECM-bound, latent transforming growth factor-β (TGF-β) complex by certain αv integrins [47], or the ability of some integrins to induce or activate matrix metalloproteases (MMPs) or other extracellular proteases that release reservoirs of ECM-associated growth factors to stimulate angiogenesis or other stromal functions [44,48,49,50].

**Figure 2:**
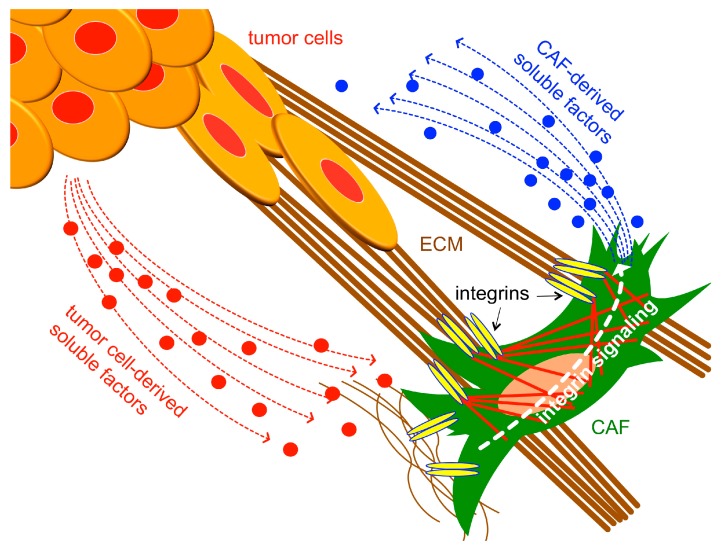
Integrins expressed on CAFs may control their ability to modulate the TME through both chemical signals and mechanical signals propagated through changes in ECM stiffness, as well as their ability to respond to chemical and mechanical cues from the TME.

**Figure 3:**
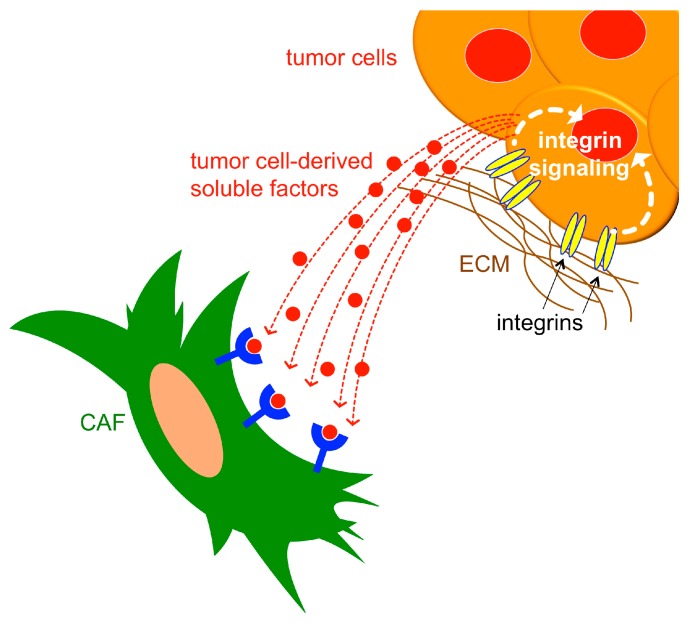
Integrins expressed on tumor cells regulate the expression/secretion of paracrine-acting factors that stimulate CAFs, in some cases, through binding to receptors expressed on the CAF surface. ECM, extracellular matrix; CAFs, cancer-associated fibroblasts.

The remainder of this review will focus on the potential roles that integrins play in regulating CAF differentiation and function, using wound fibroblasts/myofibroblasts as a model where appropriate. Below, we discuss emerging roles for specific CAF integrins in controlling their ability to generate or respond to, mechanical and biochemical cues in the TME (Figure 2). In addition, we discuss recently discovered roles of certain integrins on epithelial cells in promoting the paracrine stimulation of fibroblasts/myofibroblasts, and the potential for such intercellular crosstalk to regulate CAF differentiation and function within the TME (Figure 3). The following sub-sections are organized around recent advances towards understanding the roles that distinct integrins, or subgroups of related integrins, play in such regulation. This organization highlights the need to understand how integrins function both individually and collectively from within distinct cellular compartments of the TME to support a pro-tumorigenic niche. We will consider roles that integrins potentially play within CAFs themselves (Figure 2), as well as within tumor cells to regulate the expression/secretion of paracrine-acting factors that stimulate CAFs (Figure 3). 

### 2.1. Fibronectin-Binding Integrins

It is well established that fibroblasts differentiate into smooth muscle cell α-actin (SMC actin)-positive myofibroblasts in a process that requires sufficient ECM mechanical stiffness, active TGF-β, and an ECM in which FNs are deposited that include the alternatively spliced extra domain-A (EDA/EIIIA) domain [41,51]. In turn, these contractile myofibroblasts increase the mechanical stiffness of the ECM, produce more latent TGF-β, and deposit abundant interstitial collagens and FN, engaging a feed-forward loop that promotes additional myofibroblast differentiation. Importantly, these traits of myofibroblasts are likely to be shared by CAFs [39,52]. 

An exhaustive review of the literature yields few references on CAF integrins, which highlights an important knowledge gap in the field. Fortunately, wound fibroblasts and myofibroblasts provide a useful paradigm owing to their intensive study in recent years. Early work suggested an important role for FN isoforms that include the alternatively spliced EIIIA/EDA segment in TGF-β-dependent myofibroblast differentiation [53]. Importantly, EIIIA/EDA and EIIIB/EDB forms of FN are prominently expressed in tumor stroma [4,51,54,55], potentially implicating the integrins that bind to them. Work also focused on identifying potential receptors that bind the EIIIA/EDA segment of fibronectin, and receptors of two types have been identified: integrins (α4β1, α4β7, and α9β1) [56,57,58] and a Toll-like receptor, TLR4 [59]. Of these, integrin α4β1 is expressed on a variety of primary fibroblasts and integrin α4β7 on primary lung fibroblasts. In contrast, α9β1 is prominent on epithelial cells, neutrophils, and muscle cells and appears to not be expressed by fibroblasts [60,61]. Mice engineered to express FNs at normal levels, but that constitutively lack the EIIIA/EDA segment, exhibited fewer myofibroblasts as well as reduced fibrosis in models of lung fibrosis, allergen-induced airway fibrosis, and atherogenesis [57,62]. Although no effect on the numbers of stromal myofibroblasts in a model of pancreatic cancer has been observed [63]. However, using a mouse model of myocardial infarction, Arslan and coworkers reported reduced ECM remodeling and inflammation, with improved repair, in mice expressing FN without EIIIA/EDA [64]. Collectively, these findings suggest an important role for α4-containing integrins, together with EIIIA/EDA FNs, in regulating fibroblast and myofibroblast function. However, their role in CAFs is an area in which more study is needed.

How do α4 integrins affect changes in the fibroblast/myofibroblast phenotype? Integrin α4β1 and EIIIA/EDA-containing FNs were reported to promote an increase in FN expression, FN matrix assembly, stress fibers, and myosin light chain phosphorylation, consistent with a role in establishing a pro-fibrogenic phenotype [65]. Integrin α4β1 has also been shown to promote FN matrix assembly via the CS-1 region of FN [30]. TLR4 functions in the innate immune response in immune cells and fibroblasts, and together with EIIIA/EDA FNs induces cytokine production and promotes fibrosis in a murine cutaneous model [66,67]. Interestingly, TLR4 may also work synergistically with α4β1 to promote fibrotic gene induction [68]. Together these data suggest a role for α4 integrins in ECM remodeling and associated changes in the cytoskeleton and potentially fibrotic gene expression. Indeed, in the bleomycin murine model, the extent of lung fibrosis was reduced coincident with diminishing numbers of myofibroblasts when mice were treated with an anti-α4 antibody [69]. However, the precise mechanisms through which α4 integrins alter the myofibroblast phenotype and the extent to which these mechanisms may extend to CAFs remain unclear. 

### 2.2. Collagen-Binding Integrins

The collagen-binding integrins α1β1, α2β1, and α11β1 are expressed on fibroblasts. However, the roles of α1β1 and α2β1 in mediating critical fibroblast-mediated functions during tumorigenesis have been elusive. During wound healing, collagen deposition is a critical process that enables prompt, but imperfect, repair albeit with compromised collagen assembly. Intriguingly, while the ultrastructure of assembled collagen in excisional wounds in integrin α2-null vs. wild type mice was identical, subtle differences were observed in the tensile strength in the wounds of α2β1-deficient mice suggesting a role in collagen assembly [70]. By contrast, conspicuous roles have been observed for integrin α11β1, with reductions in the extent of granulation tissue formation and wound contraction observed in α11-null mice [71]. Indeed, integrin α11β1 is prominently expressed on fibroblasts, and α11β1 (i.e., α11 knockouts) leads to markedly reduced SMC actin expression and lower tensile strength of healed wounds [71]. Integrin α11β1 and SMC actin are both markedly upregulated in the stroma of human oral squamous cell carcinoma [72]. By breeding α11-null mice onto a severe combined immune deficiency (SCID) background, Navab and coworkers demonstrated reduced growth of human non-small cell lung carcinoma and adenocarcinoma in the mice lacking α11β1 [73]. Importantly, tumor extracts from mice lacking α11β1 had notably less collagen crosslinking and reduced tumor lysyl oxidase 1 expression. Lysyl oxidases are critical enzymes that crosslink collagen, thereby increasing the mechanical stiffness of the ECM. Because this stiff ECM is an important factor in tumor progression, these findings position integrin α11β1 as a key modulator of ECM stiffness in solid tumors and implicate it as a potential therapeutic target [74,75,76]. Collectively, these discoveries on α11β1, along with those on other fibroblast integrins (i.e., α4 integrins, see above; α3β1, and αv integrins, see below), suggest that fibroblast integrins serve diverse and sometimes subtle functions that set them apart from the original discoveries of their adhesive function. Moreover, the data suggest that there exist at least partially redundant networks that promote myofibroblast differentiation, likely in both wounds and tumors, highlighting the difficulties in targeting these overlapping pathways therapeutically to blunt myofibroblast function [51].

### 2.3. αv Integrins

Several studies have shown that members of the αv integrin subgroup have complex roles in the intercellular communication that occurs between tumor cells and CAFs, where they can function in both cell types to regulate biochemical and mechanical cues in the extracellular environment. In one study, CAF-secreted interleukin 32, an RGD motif–containing cytokine, was shown to bind integrin αvβ3 expressed on breast cancer cells to activate a p38 mitogen activated protein kinase (MAPK) signaling pathway that drives tumor cell invasion [77]. Another study used a co-culture model to identify αv integrin on carcinoma cells as a regulator of directional migration in response to CAF-mediated FN fibrillogenesis and alignment [78]. Interestingly, FN organization by CAFs required myosin-II–driven contractility and increased traction forces that were transduced to the ECM through integrin α5β1 [78], providing a clear example of how coordinated functions of different integrins expressed on CAFs and tumor cells could regulate their intercellular communication. Certain αv integrins can also stimulate cancer cell invasion from within CAFs. For example, αvβ3 was shown to be essential for CAF-mediated FN assembly that stimulated the invasion of colon carcinoma cells [79]. Finally, integrins can also regulate the ability of CAFs to stimulate inflammation in the TME that supports tumor growth and progression. Indeed, a recent study in breast cancer cells identified secreted osteopontin as a mediator of paracrine signaling to stromal fibroblasts that activates a pro-inflammatory CAF phenotype through binding to its receptors, integrin αvβ3, and CD44 [80].

As indicated above, ECM remodeling is proving to be a critical aspect of myofibroblast and CAF differentiation. This is particularly germane for TGF-β, which is stored in the wound ECM as a “large” latent complex from which active TGF-β is released in an integrin αv-dependent process. The latency-associated protein (LAP)—to which TGF-β is bound non-covalently—is covalently attached to the latent TGF-β binding protein, which is bound to fibronectin and fibulin within the ECM [45,81]. The liberation of active TGF-β from this complex can occur by several mechanisms (e.g., proteolysis; thrombospondin) depending on the cell type. Epithelial cells and many cancer cells express integrin αvβ6, which activates latent TGF-β by binding to the RGD sequence within LAP and releases active TGF-β from the latency complex [82]. Recent data suggest that this αvβ6-dependent, TGF-β activation can function as a paracrine mechanism that induces the myofibroblast phenotype in CAFs, which in turn can produce a pro-metastatic chemokine (e.g., SDF-1/CXCL12) [83,84]. Importantly, fibroblasts and myofibroblast-bearing integrins (i.e., αvβ3, αvβ5) also release TGF-β from an ECM that is mechanically stiff in a process that requires robust cell contraction [81,85]. Importantly, recent data demonstrate that EDA-containing forms of FN promote the recruitment of the large latent TGF-β complex to the ECM deposited by fibroblasts [86], potentially implicating α4β1 in this regulation (see above). Despite the central function of active TGF-β in myofibroblast and CAF differentiation, there are many processes (e.g., inflammation) mediated by this factor. Hence, targeting TGF-β safely as a therapy is not a viable option. Instead, it has recently been proposed that the ECM itself may serve as a useful therapeutic target given its role in establishing feed-forward loops that promote myofibroblast differentiation [43], an idea that further implicates its integrin receptors as potential targets.

### 2.4. Laminin-Binding Integrins

The laminin-binding integrins are emerging as important regulators of CAF function within the TME. This regulation can occur within CAFs themselves, or within tumor cells to control paracrine crosstalk with CAFs. Among the LN-binding integrins, α3β1 is the best studied in this regard as discussed below.

#### 2.4.1. Integrin α3β1 Binding to Laminin-332 Promotes CAF Differentiation and Function

Published studies have implicated LNs, sometimes in coordination with other ECM proteins, in regulating the ability of CAFs to support a pro-invasive TME. For example, α3β1 (an LN receptor) and α5β1 (an FN receptor) were each found to be required on CAFs for protease- and force-mediated ECM remodeling to generate matrix tracks that guide collective invasion of adjacent carcinoma cells, with indications that these two integrins work through distinct signaling pathways (Rho-dependent in CAFs; Cdc42-dependent in carcinoma cells) [87]. In another recent study, Cavaco and coworkers demonstrated a critical role for α3β1, and its interaction with laminin-332 (LN-332), in CAF differentiation and maintenance [88]. Using CRISPR-Cas9 to generate CAFs with a knockout of the ITGA3 gene (which encodes the α3 integrin subunit), the authors showed that α3β1 is essential for the ability of CAFs to promote invasion of pancreatic duct adenocarcinoma cells (AsPC-1) in a spheroid co-culture model. Moreover, CAFs contributed to the ectopic deposition of LN-332 in this model, and presumably to the activation of α3β1. Interestingly, the authors used a function-blocking antibody to rule out a requirement for α6 integrins (which also bind LN-332), identifying α3β1 as the primary receptor for LN-332-mediated effects on CAFs [88].

#### 2.4.2. Integrin α3β1 in Epithelial Cells Regulates the Paracrine Stimulation of Stromal Cells

Many studies over the last two decades have used genetic approaches or integrin blocking antibodies to establish a critical role for integrin α3β1 in regulating the secretion of numerous proteins by epidermal keratinocytes that modulate the skin microenvironment, including growth factors, extracellular proteases, and ECM/matricellular proteins (reviewed in [13,38]). Interestingly, α3β1-dependent induction of the genes that encode many of these proteins occurred in immortalized/transformed keratinocytes but not primary keratinocytes, suggesting that gene regulatory functions of α3β1 may be acquired by immortalized or activated keratinocytes [89,90,91]. Our mass spectrometry (MS)-based proteomic analysis of secreted proteins from wild type versus α3-null immortalized keratinocytes confirmed that α3β1 is an important regulator of the keratinocyte secretome ([92,93], and unpublished data). Consistently, a recent study from the Has group showed that the secretome is altered in keratinocytes isolated from patients with interstitial lung disease, nephrotic syndrome, and epidermolysis bullosa (ILNEB), which lack α3β1 due to inherited mutations in the ITGA3 gene [94]. Indeed, MS analysis revealed that the profile of secreted proteins was altered in ILNEB keratinocytes, including a shift towards the deposition of a FN-rich matrix and upregulation of FN-binding integrins (α5β1 and αv integrins). Collectively, the above studies show that α3β1 regulates the epidermal secretome during normal and pathological tissue remodeling.

It has long been known that growth factors and cytokines produced by the epidermal or tumor cell compartment can diffuse into the adjacent stroma to stimulate endothelial cells, fibroblasts, and immune cells [95,96,97,98]. Some keratinocyte integrins can regulate growth factor expression, leading to paracrine stimulation of stromal cells. For example, deletion of α3β1 specifically from the epidermis leads to impaired wound angiogenesis in vivo, and immortalized α3-null keratinocytes show reduced expression of at least two pro-angiogenic factors, mitogen-regulated protein 3 (MRP-3) and MMP-9 [90,99,100]. Of note, both MMP-9 and MRP family members have been linked to tumor angiogenesis [48,101,102], suggesting that α3β1 on tumor cells might similarly regulate paracrine stimulation of endothelial cells in the tumor vasculature. Strong literature supports similar crosstalk from keratinocytes to stromal fibroblasts in both tumors and wounds, as reviewed elsewhere [37,39,103], and co-culture studies have demonstrated that keratinocyte-derived growth factors, ECM proteins, and MMPs can induce gene expression programs in fibroblasts [104]. 

Although roles for specific integrins in controlling paracrine signaling from tumor cells to CAFs are only beginning to be explored, recent findings of α3β1-dependent crosstalk from keratinocytes to dermal fibroblasts in the wound microenvironment may offer clues. Indeed, we recently identified a novel role for epidermal α3β1 in the crosstalk from keratinocytes to dermal fibroblasts that regulates the differentiated state [93]. Specifically, we showed that α3β1 is required for paracrine signaling from keratinocytes that stimulates Cox-2 expression/PGE_2_ signaling and suppresses TGF-β-induced α-SMA expression in fibroblasts, and that this effect is mediated through α3β1-dependent production by keratinocytes of interleukin-1α (IL-1α) [93]. Importantly, IL-1α has been shown to regulate myofibroblast differentiation in keratinocyte-fibroblast co-cultures, implicating this cytokine in the temporal control of myofibroblast differentiation during wound healing [105]. Interestingly, tumor cell-associated IL-1α can also mediate the paracrine stimulation of CAFs to induce a pro-inflammatory phenotype in a co-culture model of pancreatic ductal adenocarcinoma [106,107]. Ongoing studies in our group are exploring whether α3β1 on tumor cells influences CAF differentiation or function within the TME, through the secretion of IL-1α or other paracrine factors. 

## 3. Conclusions

Given the distinct roles that individual integrins have been shown to play in regulating the differentiation and function of myofibroblasts or CAFs, and the importance of intercellular communication to maintain a tumor-supportive stroma, it is becoming increasingly clear that a complete understanding of integrin control over the TME must take into consideration the combined roles of individual integrins within distinct cell types of the tumor and its stroma. Indeed, this mind-set will almost certainly be essential as the field moves towards developing effective combinatorial strategies to target integrins in the cancer clinic. While there is still much to learn about the complex roles that integrins play in CAFs, it is instructive to consider the emerging paradigms for integrin regulation of myofibroblasts in wound healing or fibrotic conditions, as it seems likely that similar regulations will also apply to CAFs in the TME.
